# Supplementary material for: A Transcription Regulatory Sequence in the 5′ Untranslated Region of SARS-CoV-2 Is Vital for Virus Replication with an Altered Evolutionary Pattern against Human Inhibitory MicroRNAs
Source: Cells. 2021 Feb 4;10(2):319. doi: 10.3390/cells10020319 (PMC7913991; doi:10.3390/cells10020319)
Supplement: Supplementary file 1 [file cells-10-00319-s001.zip › Supplementary files/Supplementary 2 Multivariate analysis.docx]

**Supplementary 1**. Multivariate analysis. Multivariate analysis of thermodynamic binding energy values of leader sequence of coronaviruses against 39 mined microRNAs (Table 3 and Figure 6 of the paper).

Contents

[(A) Principle Component Analysis discriminates SARS-CoV-2 from the rest of coronaviruses. 1](#_Toc51536102)

[Eigenanalysis of the Correlation Matrix 1](#_Toc51536103)

[Eigenvectors 1](#_Toc51536104)

[Important microRNAs in PCA1 (feature selection) 6](#_Toc51536105)

[Important microRNAs in PCA2 (feature selection) 7](#_Toc51536106)

[(B) Clustering 8](#_Toc51536107)

[Amalgamation Steps 8](#_Toc51536108)

# (A) Principle Component Analysis discriminates SARS-CoV-2 from the rest of coronaviruses.

## Eigenanalysis of the Correlation Matrix

| Eigenvalue | 18.761 | | | 9.280 | | 5.622 | | | 5.336 | | | 0.000 | | | 0.000 | | 0.000 | | | 0.000 | | | 0.000 | | | 0.000 | | | 0.000 | | |
| --- | --- | --- | --- | --- | --- | --- | --- | --- | --- | --- | --- | --- | --- | --- | --- | --- | --- | --- | --- | --- | --- | --- | --- | --- | --- | --- | --- | --- | --- | --- | --- |
| Proportion | 0.481 | | | 0.238 | | 0.144 | | | 0.137 | | | 0.000 | | | 0.000 | | 0.000 | | | 0.000 | | | 0.000 | | | 0.000 | | | 0.000 | | |
| Cumulative | 0.481 | | | 0.719 | | 0.863 | | | 1.000 | | | 1.000 | | | 1.000 | | 1.000 | | | 1.000 | | | 1.000 | | | 1.000 | | | 1.000 | | |
| Eigenvalue | 0.000 | 0.000 | | | 0.000 | | | 0.000 | | | -0.000 | | | -0.000 | | | | -0.000 | | | -0.000 | | | -0.000 | | | -0.000 | | |  |  |
| Proportion | 0.000 | 0.000 | | | 0.000 | | | 0.000 | | | -0.000 | | | -0.000 | | | | -0.000 | | | -0.000 | | | -0.000 | | | -0.000 | | |  |  |
| Cumulative | 1.000 | 1.000 | | | 1.000 | | | 1.000 | | | 1.000 | | | 1.000 | | | | 1.000 | | | 1.000 | | | 1.000 | | | 1.000 | | |  |  |
| Eigenvalue | -0.000 | | -0.000 | | | | -0.000 | | | -0.000 | | | -0.000 | | | -0.000 | | | -0.000 | | | -0.000 | | | -0.000 | | | -0.000 | | |  |
| Proportion | -0.000 | | -0.000 | | | | -0.000 | | | -0.000 | | | -0.000 | | | -0.000 | | | -0.000 | | | -0.000 | | | -0.000 | | | -0.000 | | |  |
| Cumulative | 1.000 | | 1.000 | | | | 1.000 | | | 1.000 | | | 1.000 | | | 1.000 | | | 1.000 | | | 1.000 | | | 1.000 | | | 1.000 | | |  |
| Eigenvalue | -0.000 | | -0.000 | | | | -0.000 | | | -0.000 | | | -0.000 | | | -0.000 | | | -0.000 | | | -0.000 | | |  |  |  |  |  |  |  |
| Proportion | -0.000 | | -0.000 | | | | -0.000 | | | -0.000 | | | -0.000 | | | -0.000 | | | -0.000 | | | -0.000 | | |  |  |  |  |  |  |  |
| Cumulative | 1.000 | | 1.000 | | | | 1.000 | | | 1.000 | | | 1.000 | | | 1.000 | | | 1.000 | | | 1.000 | | |  |  |  |  |  |  |  |

## Eigenvectors

| **Variable** | **PC1** | **PC2** | | **PC3** | | **PC4** | | **PC5** | | **PC6** | | **PC7** | | **PC8** | | **PC9** | |  |
| --- | --- | --- | --- | --- | --- | --- | --- | --- | --- | --- | --- | --- | --- | --- | --- | --- | --- | --- |
| ssc-miR-9833-5p | 0.167 | 0.093 | | -0.221 | | 0.149 | | -0.120 | | 0.024 | | 0.192 | | -0.297 | | 0.155 | |  |
| ptc-miR474b | 0.188 | 0.167 | | -0.102 | | 0.065 | | -0.023 | | 0.249 | | -0.070 | | -0.259 | | -0.096 | |  |
| ptc-miR474a | 0.128 | 0.224 | | -0.145 | | 0.143 | | 0.183 | | -0.025 | | 0.032 | | -0.221 | | 0.102 | |  |
| csa-let-7d | 0.067 | 0.314 | | -0.015 | | 0.025 | | -0.143 | | 0.101 | | 0.273 | | 0.385 | | -0.115 | |  |
| cin-let-7d-5p | 0.067 | 0.314 | | -0.015 | | 0.025 | | -0.143 | | 0.101 | | 0.273 | | 0.385 | | -0.115 | |  |
| mtr-miR2629g | 0.198 | -0.121 | | 0.100 | | -0.119 | | 0.030 | | 0.009 | | 0.160 | | -0.056 | | 0.024 | |  |
| mtr-miR2629f | 0.198 | -0.121 | | 0.100 | | -0.119 | | 0.030 | | 0.009 | | 0.160 | | -0.056 | | 0.024 | |  |
| mtr-miR2629e | 0.198 | -0.121 | | 0.100 | | -0.119 | | 0.030 | | 0.009 | | 0.160 | | -0.056 | | 0.024 | |  |
| mtr-miR2629d | 0.198 | -0.121 | | 0.100 | | -0.119 | | 0.030 | | 0.009 | | 0.160 | | -0.056 | | 0.024 | |  |
| mtr-miR2629c | 0.198 | -0.121 | | 0.100 | | -0.119 | | 0.030 | | 0.009 | | 0.160 | | -0.056 | | 0.024 | |  |
| mtr-miR2629b | 0.198 | -0.121 | | 0.100 | | -0.119 | | 0.030 | | 0.009 | | 0.160 | | -0.056 | | 0.024 | |  |
| mtr-miR2629a | 0.198 | -0.121 | | 0.100 | | -0.119 | | 0.030 | | 0.009 | | 0.160 | | -0.056 | | 0.024 | |  |
| hsa-miR-5004-3p | 0.210 | 0.031 | | 0.119 | | -0.124 | | -0.309 | | 0.052 | | -0.399 | | 0.055 | | 0.158 | |  |
| mdo-miR-137b-5p | 0.181 | -0.085 | | 0.235 | | -0.036 | | -0.174 | | 0.135 | | -0.087 | | 0.158 | | -0.106 | |  |
| bta-miR-2284ab | 0.171 | 0.152 | | -0.004 | | -0.209 | | 0.025 | | 0.041 | | -0.054 | | 0.167 | | 0.601 | |  |
| oan-miR-1395-5p | 0.215 | -0.009 | | 0.139 | | -0.068 | | 0.077 | | 0.122 | | -0.035 | | 0.319 | | -0.087 | |  |
| eca-miR-9080 | 0.226 | -0.060 | | -0.022 | | -0.026 | | -0.129 | | -0.025 | | 0.135 | | -0.142 | | -0.200 | |  |
| cel-miR-2211-5p | -0.086 | -0.287 | | -0.083 | | 0.105 | | 0.052 | | 0.272 | | 0.157 | | -0.166 | | 0.032 | |  |
| bdi-miR5065 | -0.181 | 0.128 | | 0.180 | | -0.098 | | 0.084 | | -0.067 | | 0.063 | | 0.000 | | 0.043 | |  |
| dsi-miR-986-3p | -0.170 | 0.103 | | 0.219 | | -0.131 | | 0.009 | | 0.185 | | 0.062 | | -0.151 | | -0.013 | |  |
| dme-miR-986-3p | -0.170 | 0.103 | | 0.219 | | -0.131 | | 0.009 | | 0.185 | | 0.062 | | -0.151 | | -0.013 | |  |
| ath-miR5638a | -0.181 | -0.183 | | -0.082 | | 0.079 | | 0.051 | | 0.003 | | -0.021 | | 0.232 | | 0.042 | |  |
| ame-miR-3741 | -0.030 | 0.062 | | 0.274 | | 0.314 | | -0.089 | | -0.220 | | 0.016 | | -0.075 | | 0.272 | |  |
| ppy-miR-1273a | 0.040 | 0.069 | | 0.330 | | 0.243 | | -0.095 | | -0.163 | | -0.034 | | -0.019 | | 0.254 | |  |
| dps-miR-2535-3p | -0.217 | -0.068 | | 0.103 | | -0.049 | | -0.387 | | 0.318 | | -0.105 | | -0.106 | | -0.192 | |  |
| bdi-miR5065_1 | -0.181 | 0.128 | | 0.180 | | -0.098 | | 0.085 | | -0.033 | | 0.058 | | -0.029 | | 0.002 | |  |
| mghv-miR-M1-2-3p | 0.163 | -0.046 | | 0.023 | | 0.300 | | 0.125 | | 0.252 | | 0.060 | | 0.112 | | 0.084 | |  |
| dsi-miR-986-3p_1 | -0.170 | 0.103 | | 0.219 | | -0.131 | | 0.006 | | 0.155 | | 0.056 | | -0.150 | | 0.112 | |  |
| dme-miR-986-3p_1 | -0.170 | 0.103 | | 0.219 | | -0.131 | | 0.006 | | 0.155 | | 0.056 | | -0.150 | | 0.112 | |  |
| gga-miR-6608-3p | 0.093 | 0.096 | | 0.206 | | 0.310 | | 0.055 | | 0.421 | | -0.163 | | -0.029 | | -0.068 | |  |
| csi-miR3953 | 0.096 | 0.075 | | 0.227 | | 0.302 | | -0.116 | | -0.260 | | 0.067 | | -0.146 | | -0.044 | |  |
| cel-miR-8207-3p | 0.039 | -0.121 | | 0.218 | | 0.326 | | -0.007 | | -0.096 | | 0.052 | | 0.035 | | -0.202 | |  |
| bmo-miR-3293 | -0.032 | -0.204 | | 0.219 | | 0.246 | | 0.278 | | 0.131 | | 0.028 | | 0.162 | | -0.001 | |  |
| mmu-miR-6957-3p | 0.117 | -0.099 | | 0.260 | | -0.227 | | 0.079 | | -0.198 | | -0.361 | | 0.048 | | -0.123 | |  |
| ppc-miR-83-5p | -0.018 | -0.325 | | -0.003 | | -0.054 | | 0.115 | | 0.145 | | 0.062 | | 0.028 | | 0.054 | |  |
| ptc-miR6464 | 0.208 | 0.070 | | -0.141 | | 0.079 | | 0.271 | | 0.213 | | -0.435 | | -0.052 | | 0.045 | |  |
| oan-miR-1421l-2-3p | -0.142 | -0.255 | | 0.010 | | 0.057 | | 0.080 | | -0.171 | | -0.063 | | 0.073 | | -0.052 | |  |
| dme-miR-4949-3p | -0.009 | -0.303 | | -0.128 | | 0.100 | | -0.596 | | 0.061 | | -0.034 | | 0.022 | | 0.206 | |  |
| cme-miR1863 | -0.198 | -0.160 | | -0.044 | | 0.054 | | 0.068 | | 0.182 | | 0.116 | | 0.189 | | 0.394 | |  |
| **Variable** | **PC10** | | **PC11** | | **PC12** | | **PC13** | | **PC14** | | **PC15** | | **PC16** | | **PC17** | | **PC18** | |
| ssc-miR-9833-5p | -0.168 | | -0.056 | | -0.076 | | -0.277 | | 0.273 | | 0.422 | | 0.193 | | -0.086 | | 0.078 | |
| ptc-miR474b | 0.101 | | -0.065 | | 0.215 | | 0.218 | | -0.098 | | 0.322 | | 0.040 | | 0.055 | | -0.152 | |
| ptc-miR474a | -0.046 | | 0.137 | | -0.112 | | 0.511 | | 0.397 | | -0.131 | | -0.221 | | -0.079 | | 0.248 | |
| csa-let-7d | -0.002 | | -0.037 | | -0.227 | | 0.128 | | 0.008 | | 0.026 | | -0.012 | | -0.042 | | -0.001 | |
| cin-let-7d-5p | -0.002 | | -0.037 | | -0.227 | | 0.128 | | 0.008 | | 0.026 | | -0.012 | | -0.042 | | -0.001 | |
| mtr-miR2629g | 0.007 | | -0.060 | | -0.012 | | 0.039 | | 0.040 | | -0.029 | | -0.050 | | 0.094 | | 0.010 | |
| mtr-miR2629f | 0.007 | | -0.060 | | -0.012 | | 0.039 | | 0.040 | | -0.029 | | -0.050 | | 0.094 | | 0.010 | |
| mtr-miR2629e | 0.007 | | -0.060 | | -0.012 | | 0.039 | | 0.040 | | -0.029 | | -0.050 | | 0.094 | | 0.010 | |
| mtr-miR2629d | 0.007 | | -0.060 | | -0.012 | | 0.039 | | 0.040 | | -0.029 | | -0.050 | | 0.094 | | 0.010 | |
| mtr-miR2629c | 0.007 | | -0.060 | | -0.012 | | 0.039 | | 0.040 | | -0.029 | | -0.050 | | 0.094 | | 0.010 | |
| mtr-miR2629b | 0.007 | | -0.060 | | -0.012 | | 0.039 | | 0.040 | | -0.029 | | -0.050 | | 0.094 | | 0.010 | |
| mtr-miR2629a | 0.007 | | -0.060 | | -0.012 | | 0.039 | | 0.040 | | -0.029 | | -0.050 | | 0.094 | | 0.010 | |
| hsa-miR-5004-3p | 0.095 | | 0.314 | | -0.312 | | -0.120 | | 0.245 | | -0.302 | | 0.038 | | 0.198 | | 0.016 | |
| mdo-miR-137b-5p | 0.016 | | 0.373 | | 0.288 | | -0.082 | | 0.223 | | 0.201 | | -0.024 | | -0.335 | | 0.214 | |
| bta-miR-2284ab | -0.301 | | -0.016 | | 0.252 | | 0.106 | | -0.330 | | -0.069 | | -0.055 | | -0.311 | | -0.057 | |
| oan-miR-1395-5p | 0.024 | | -0.017 | | 0.350 | | -0.004 | | -0.047 | | 0.098 | | 0.293 | | 0.230 | | 0.266 | |
| eca-miR-9080 | 0.162 | | 0.085 | | -0.042 | | -0.171 | | -0.180 | | -0.043 | | -0.254 | | -0.567 | | -0.153 | |
| cel-miR-2211-5p | -0.149 | | 0.435 | | -0.219 | | 0.199 | | -0.451 | | -0.069 | | 0.085 | | 0.030 | | 0.252 | |
| bdi-miR5065 | 0.054 | | 0.182 | | 0.065 | | -0.138 | | 0.090 | | -0.019 | | -0.055 | | 0.000 | | -0.015 | |
| dsi-miR-986-3p | -0.057 | | 0.083 | | 0.192 | | 0.041 | | 0.160 | | -0.018 | | -0.092 | | -0.001 | | -0.071 | |
| dme-miR-986-3p | -0.057 | | 0.083 | | 0.192 | | 0.041 | | 0.160 | | -0.018 | | -0.092 | | -0.001 | | -0.071 | |
| ath-miR5638a | -0.010 | | 0.265 | | 0.088 | | 0.073 | | 0.032 | | 0.375 | | -0.292 | | 0.190 | | 0.003 | |
| ame-miR-3741 | 0.376 | | 0.046 | | -0.019 | | 0.106 | | -0.115 | | 0.246 | | 0.006 | | -0.007 | | 0.357 | |
| ppy-miR-1273a | 0.221 | | -0.290 | | 0.014 | | 0.055 | | -0.094 | | -0.035 | | -0.053 | | -0.007 | | -0.014 | |
| dps-miR-2535-3p | 0.083 | | -0.258 | | 0.109 | | 0.436 | | -0.011 | | -0.080 | | 0.110 | | -0.079 | | -0.004 | |
| bdi-miR5065_1 | 0.111 | | 0.113 | | -0.043 | | -0.005 | | -0.071 | | -0.061 | | -0.356 | | -0.049 | | 0.001 | |
| mghv-miR-M1-2-3p | 0.327 | | 0.235 | | -0.003 | | 0.012 | | 0.047 | | 0.050 | | -0.003 | | 0.068 | | -0.577 | |
| dsi-miR-986-3p_1 | -0.029 | | -0.001 | | -0.203 | | -0.130 | | 0.021 | | 0.108 | | 0.203 | | -0.050 | | -0.041 | |
| dme-miR-986-3p_1 | -0.029 | | -0.001 | | -0.203 | | -0.130 | | 0.021 | | 0.108 | | 0.203 | | -0.050 | | -0.041 | |
| gga-miR-6608-3p | -0.203 | | -0.241 | | -0.110 | | -0.302 | | -0.179 | | -0.005 | | -0.437 | | 0.184 | | 0.146 | |
| csi-miR3953 | -0.249 | | 0.225 | | 0.148 | | 0.125 | | -0.118 | | -0.157 | | 0.213 | | 0.161 | | -0.355 | |
| cel-miR-8207-3p | -0.467 | | -0.017 | | 0.051 | | -0.040 | | 0.142 | | -0.060 | | -0.063 | | -0.072 | | 0.029 | |
| bmo-miR-3293 | -0.019 | | -0.076 | | -0.155 | | 0.046 | | 0.127 | | -0.153 | | 0.348 | | -0.362 | | 0.060 | |
| mmu-miR-6957-3p | -0.228 | | -0.029 | | -0.389 | | 0.291 | | -0.081 | | 0.483 | | -0.048 | | -0.064 | | -0.194 | |
| ppc-miR-83-5p | 0.264 | | -0.076 | | -0.105 | | -0.015 | | -0.000 | | 0.040 | | 0.014 | | -0.048 | | 0.008 | |
| ptc-miR6464 | 0.050 | | -0.069 | | 0.010 | | 0.020 | | 0.077 | | -0.099 | | 0.047 | | -0.121 | | -0.003 | |
| oan-miR-1421l-2-3p | 0.045 | | -0.185 | | 0.096 | | 0.056 | | 0.194 | | -0.024 | | -0.139 | | -0.147 | | -0.054 | |
| dme-miR-4949-3p | -0.092 | | -0.066 | | -0.026 | | 0.011 | | 0.071 | | -0.005 | | -0.110 | | -0.014 | | -0.032 | |
| cme-miR1863 | -0.162 | | -0.094 | | -0.013 | | 0.067 | | 0.248 | | 0.070 | | -0.068 | | 0.035 | | -0.187 | |
| **Variable** | **PC19** | | **PC20** | | **PC21** | | **PC22** | | **PC23** | | **PC24** | | **PC25** | | **PC26** | | **PC27** | |
| ssc-miR-9833-5p | 0.045 | | -0.168 | | -0.205 | | -0.031 | | 0.000 | | 0.000 | | -0.000 | | 0.000 | | -0.000 | |
| ptc-miR474b | 0.160 | | 0.133 | | 0.066 | | 0.087 | | -0.000 | | -0.000 | | 0.000 | | -0.000 | | 0.000 | |
| ptc-miR474a | -0.056 | | 0.107 | | 0.176 | | -0.084 | | 0.000 | | 0.000 | | -0.000 | | 0.000 | | -0.000 | |
| csa-let-7d | 0.048 | | -0.027 | | -0.025 | | 0.068 | | -0.000 | | -0.000 | | 0.000 | | -0.500 | | -0.500 | |
| cin-let-7d-5p | 0.048 | | -0.027 | | -0.025 | | 0.068 | | -0.000 | | -0.000 | | 0.000 | | 0.500 | | 0.500 | |
| mtr-miR2629g | -0.021 | | 0.045 | | 0.004 | | 0.034 | | -0.092 | | -0.089 | | -0.908 | | -0.026 | | 0.026 | |
| mtr-miR2629f | -0.021 | | 0.045 | | 0.004 | | 0.034 | | -0.092 | | 0.910 | | 0.092 | | -0.026 | | 0.026 | |
| mtr-miR2629e | -0.021 | | 0.045 | | 0.004 | | 0.034 | | -0.092 | | -0.122 | | 0.093 | | -0.026 | | 0.026 | |
| mtr-miR2629d | -0.021 | | 0.045 | | 0.004 | | 0.034 | | 0.908 | | -0.089 | | 0.092 | | -0.026 | | 0.026 | |
| mtr-miR2629c | -0.021 | | 0.045 | | 0.004 | | 0.034 | | -0.300 | | -0.290 | | 0.299 | | 0.097 | | -0.097 | |
| mtr-miR2629b | -0.021 | | 0.045 | | 0.004 | | 0.034 | | -0.150 | | -0.145 | | 0.150 | | 0.217 | | -0.217 | |
| mtr-miR2629a | -0.021 | | 0.045 | | 0.004 | | 0.034 | | -0.182 | | -0.176 | | 0.181 | | -0.210 | | 0.210 | |
| hsa-miR-5004-3p | 0.130 | | 0.043 | | -0.148 | | 0.153 | | -0.000 | | -0.000 | | 0.000 | | -0.000 | | 0.000 | |
| mdo-miR-137b-5p | -0.051 | | 0.002 | | 0.199 | | -0.324 | | 0.000 | | 0.000 | | -0.000 | | 0.000 | | -0.000 | |
| bta-miR-2284ab | -0.085 | | 0.020 | | -0.002 | | 0.031 | | 0.000 | | 0.000 | | -0.000 | | 0.000 | | -0.000 | |
| oan-miR-1395-5p | 0.042 | | 0.057 | | -0.081 | | -0.042 | | 0.000 | | 0.000 | | -0.000 | | -0.000 | | 0.000 | |
| eca-miR-9080 | 0.078 | | 0.161 | | -0.072 | | 0.298 | | -0.000 | | -0.000 | | 0.000 | | 0.000 | | -0.000 | |
| cel-miR-2211-5p | 0.204 | | -0.183 | | 0.070 | | -0.044 | | 0.000 | | 0.000 | | -0.000 | | 0.000 | | -0.000 | |
| bdi-miR5065 | -0.086 | | -0.206 | | 0.584 | | 0.381 | | -0.000 | | -0.000 | | 0.000 | | -0.000 | | 0.000 | |
| dsi-miR-986-3p | 0.107 | | -0.283 | | -0.264 | | 0.162 | | -0.031 | | -0.030 | | 0.031 | | -0.107 | | 0.107 | |
| dme-miR-986-3p | 0.107 | | -0.283 | | -0.264 | | 0.162 | | 0.031 | | 0.030 | | -0.031 | | 0.107 | | -0.107 | |
| ath-miR5638a | 0.245 | | 0.434 | | -0.131 | | 0.350 | | -0.000 | | -0.000 | | 0.000 | | 0.000 | | -0.000 | |
| ame-miR-3741 | -0.342 | | -0.039 | | -0.153 | | 0.186 | | -0.000 | | -0.000 | | 0.000 | | -0.000 | | 0.000 | |
| ppy-miR-1273a | 0.663 | | -0.105 | | 0.153 | | -0.116 | | 0.000 | | 0.000 | | -0.000 | | 0.000 | | -0.000 | |
| dps-miR-2535-3p | -0.136 | | 0.090 | | 0.046 | | 0.032 | | -0.000 | | -0.000 | | 0.000 | | -0.000 | | 0.000 | |
| bdi-miR5065_1 | -0.010 | | 0.151 | | -0.381 | | -0.492 | | 0.000 | | 0.000 | | -0.000 | | 0.000 | | -0.000 | |
| mghv-miR-M1-2-3p | -0.134 | | -0.147 | | 0.110 | | -0.218 | | 0.000 | | 0.000 | | -0.000 | | 0.000 | | -0.000 | |
| dsi-miR-986-3p_1 | 0.016 | | 0.369 | | 0.143 | | -0.081 | | 0.039 | | 0.037 | | -0.039 | | 0.432 | | -0.432 | |
| dme-miR-986-3p_1 | 0.016 | | 0.369 | | 0.143 | | -0.081 | | -0.039 | | -0.037 | | 0.039 | | -0.432 | | 0.432 | |
| gga-miR-6608-3p | -0.267 | | -0.011 | | 0.062 | | 0.053 | | -0.000 | | -0.000 | | 0.000 | | -0.000 | | 0.000 | |
| csi-miR3953 | -0.189 | | 0.195 | | -0.121 | | 0.067 | | -0.000 | | -0.000 | | 0.000 | | -0.000 | | 0.000 | |
| cel-miR-8207-3p | 0.243 | | 0.040 | | 0.108 | | -0.072 | | 0.000 | | 0.000 | | -0.000 | | -0.000 | | 0.000 | |
| bmo-miR-3293 | -0.055 | | 0.020 | | -0.247 | | 0.207 | | -0.000 | | -0.000 | | 0.000 | | -0.000 | | 0.000 | |
| mmu-miR-6957-3p | -0.068 | | -0.247 | | -0.005 | | -0.020 | | 0.000 | | 0.000 | | -0.000 | | 0.000 | | -0.000 | |
| ppc-miR-83-5p | 0.036 | | -0.105 | | 0.010 | | -0.049 | | 0.000 | | 0.000 | | -0.000 | | 0.000 | | -0.000 | |
| ptc-miR6464 | 0.118 | | 0.104 | | -0.066 | | 0.123 | | -0.000 | | -0.000 | | 0.000 | | -0.000 | | 0.000 | |
| oan-miR-1421l-2-3p | -0.082 | | 0.092 | | 0.047 | | 0.002 | | -0.000 | | 0.000 | | -0.000 | | -0.000 | | 0.000 | |
| dme-miR-4949-3p | -0.075 | | -0.036 | | 0.029 | | -0.027 | | 0.000 | | -0.000 | | 0.000 | | 0.000 | | -0.000 | |
| cme-miR1863 | -0.034 | | 0.011 | | -0.004 | | -0.000 | | 0.000 | | 0.000 | | 0.000 | | 0.000 | | 0.000 | |
| **Variable** | **PC28** | | **PC29** | | **PC30** | | **PC31** | | **PC32** | | **PC33** | | **PC34** | | **PC35** | | **PC36** | |
| ssc-miR-9833-5p | -0.000 | | -0.000 | | 0.000 | | 0.000 | | -0.005 | | -0.228 | | -0.037 | | 0.122 | | 0.327 | |
| ptc-miR474b | -0.000 | | -0.000 | | -0.000 | | -0.000 | | 0.125 | | 0.496 | | 0.100 | | 0.407 | | -0.068 | |
| ptc-miR474a | -0.000 | | 0.000 | | 0.000 | | -0.000 | | -0.086 | | -0.290 | | 0.039 | | 0.129 | | -0.171 | |
| csa-let-7d | -0.000 | | 0.000 | | 0.000 | | -0.000 | | 0.067 | | 0.053 | | 0.034 | | -0.051 | | -0.002 | |
| cin-let-7d-5p | 0.000 | | -0.000 | | -0.000 | | 0.000 | | 0.067 | | 0.053 | | 0.034 | | -0.051 | | -0.002 | |
| mtr-miR2629g | -0.030 | | 0.072 | | -0.069 | | 0.064 | | 0.029 | | 0.043 | | -0.030 | | -0.055 | | 0.070 | |
| mtr-miR2629f | -0.030 | | 0.072 | | -0.046 | | 0.042 | | 0.029 | | 0.043 | | -0.030 | | -0.055 | | 0.070 | |
| mtr-miR2629e | -0.030 | | 0.072 | | 0.637 | | -0.642 | | 0.029 | | 0.043 | | -0.030 | | -0.055 | | 0.070 | |
| mtr-miR2629d | -0.030 | | 0.072 | | -0.069 | | 0.065 | | 0.029 | | 0.043 | | -0.030 | | -0.055 | | 0.070 | |
| mtr-miR2629c | -0.087 | | 0.684 | | -0.180 | | 0.258 | | 0.029 | | 0.043 | | -0.030 | | -0.055 | | 0.070 | |
| mtr-miR2629b | 0.198 | | -0.456 | | -0.571 | | -0.352 | | 0.029 | | 0.043 | | -0.030 | | -0.055 | | 0.070 | |
| mtr-miR2629a | 0.010 | | -0.515 | | 0.298 | | 0.563 | | 0.029 | | 0.043 | | -0.030 | | -0.055 | | 0.070 | |
| hsa-miR-5004-3p | 0.000 | | -0.000 | | -0.000 | | 0.000 | | -0.067 | | 0.110 | | -0.041 | | 0.247 | | 0.004 | |
| mdo-miR-137b-5p | -0.000 | | 0.000 | | 0.000 | | -0.000 | | 0.371 | | 0.061 | | 0.026 | | -0.179 | | -0.042 | |
| bta-miR-2284ab | -0.000 | | -0.000 | | 0.000 | | 0.000 | | -0.033 | | -0.041 | | -0.273 | | 0.148 | | 0.004 | |
| oan-miR-1395-5p | 0.000 | | 0.000 | | -0.000 | | -0.000 | | -0.434 | | -0.306 | | 0.333 | | 0.183 | | 0.003 | |
| eca-miR-9080 | -0.000 | | -0.000 | | 0.000 | | 0.000 | | -0.276 | | -0.151 | | 0.270 | | -0.002 | | -0.098 | |
| cel-miR-2211-5p | -0.000 | | 0.000 | | 0.000 | | 0.000 | | -0.031 | | 0.027 | | 0.132 | | -0.078 | | 0.140 | |
| bdi-miR5065 | 0.000 | | 0.000 | | -0.000 | | -0.000 | | -0.019 | | 0.014 | | 0.154 | | 0.253 | | 0.370 | |
| dsi-miR-986-3p | -0.658 | | -0.093 | | -0.148 | | -0.103 | | -0.029 | | -0.042 | | -0.030 | | -0.099 | | -0.150 | |
| dme-miR-986-3p | 0.658 | | 0.093 | | 0.148 | | 0.103 | | -0.029 | | -0.042 | | -0.030 | | -0.099 | | -0.150 | |
| ath-miR5638a | -0.000 | | -0.000 | | 0.000 | | 0.000 | | 0.108 | | -0.201 | | -0.237 | | -0.020 | | 0.103 | |
| ame-miR-3741 | -0.000 | | -0.000 | | -0.000 | | 0.000 | | -0.198 | | 0.256 | | -0.035 | | -0.161 | | -0.099 | |
| ppy-miR-1273a | -0.000 | | 0.000 | | 0.000 | | 0.000 | | 0.154 | | -0.197 | | 0.070 | | -0.058 | | 0.069 | |
| dps-miR-2535-3p | 0.000 | | 0.000 | | -0.000 | | -0.000 | | -0.092 | | -0.164 | | -0.157 | | -0.014 | | 0.287 | |
| bdi-miR5065_1 | -0.000 | | 0.000 | | 0.000 | | 0.000 | | -0.048 | | 0.116 | | 0.167 | | 0.278 | | 0.381 | |
| mghv-miR-M1-2-3p | 0.000 | | 0.000 | | 0.000 | | -0.000 | | -0.271 | | -0.107 | | -0.260 | | -0.066 | | 0.092 | |
| dsi-miR-986-3p_1 | -0.203 | | -0.102 | | 0.213 | | 0.157 | | -0.094 | | -0.045 | | -0.018 | | -0.078 | | -0.114 | |
| dme-miR-986-3p_1 | 0.203 | | 0.102 | | -0.213 | | -0.157 | | -0.094 | | -0.045 | | -0.018 | | -0.078 | | -0.114 | |
| gga-miR-6608-3p | 0.000 | | -0.000 | | 0.000 | | 0.000 | | 0.143 | | -0.166 | | 0.028 | | 0.068 | | -0.050 | |
| csi-miR3953 | 0.000 | | -0.000 | | -0.000 | | -0.000 | | 0.297 | | -0.192 | | 0.267 | | 0.016 | | -0.064 | |
| cel-miR-8207-3p | 0.000 | | 0.000 | | -0.000 | | -0.000 | | -0.426 | | 0.323 | | -0.252 | | 0.074 | | -0.098 | |
| bmo-miR-3293 | -0.000 | | -0.000 | | -0.000 | | 0.000 | | 0.225 | | 0.041 | | -0.095 | | 0.254 | | 0.174 | |
| mmu-miR-6957-3p | -0.000 | | -0.000 | | 0.000 | | 0.000 | | -0.056 | | -0.093 | | 0.093 | | 0.007 | | 0.009 | |
| ppc-miR-83-5p | -0.000 | | 0.000 | | 0.000 | | 0.000 | | 0.155 | | -0.149 | | -0.007 | | 0.363 | | -0.465 | |
| ptc-miR6464 | -0.000 | | -0.000 | | -0.000 | | 0.000 | | -0.007 | | 0.098 | | 0.181 | | -0.436 | | 0.204 | |
| oan-miR-1421l-2-3p | -0.000 | | 0.000 | | -0.000 | | -0.000 | | -0.027 | | -0.006 | | 0.080 | | 0.071 | | 0.119 | |
| dme-miR-4949-3p | 0.000 | | 0.000 | | 0.000 | | 0.000 | | -0.047 | | -0.012 | | 0.126 | | 0.040 | | 0.001 | |
| cme-miR1863 | 0.000 | | 0.000 | | 0.000 | | 0.000 | | -0.058 | | 0.223 | | 0.533 | | -0.108 | | -0.074 | |
| **Variable** | **PC37** | | **PC38** | | **PC39** | |  |  |  |  |  |  |  |  |  |  |  |  |
| ssc-miR-9833-5p | -0.184 | | -0.007 | | 0.170 | |  |  |  |  |  |  |  |  |  |  |  |  |
| ptc-miR474b | 0.101 | | -0.140 | | -0.046 | |  |  |  |  |  |  |  |  |  |  |  |  |
| ptc-miR474a | 0.119 | | 0.007 | | -0.078 | |  |  |  |  |  |  |  |  |  |  |  |  |
| csa-let-7d | -0.164 | | -0.103 | | -0.034 | |  |  |  |  |  |  |  |  |  |  |  |  |
| cin-let-7d-5p | -0.164 | | -0.103 | | -0.034 | |  |  |  |  |  |  |  |  |  |  |  |  |
| mtr-miR2629g | -0.007 | | 0.003 | | -0.012 | |  |  |  |  |  |  |  |  |  |  |  |  |
| mtr-miR2629f | -0.007 | | 0.003 | | -0.012 | |  |  |  |  |  |  |  |  |  |  |  |  |
| mtr-miR2629e | -0.007 | | 0.003 | | -0.012 | |  |  |  |  |  |  |  |  |  |  |  |  |
| mtr-miR2629d | -0.007 | | 0.003 | | -0.012 | |  |  |  |  |  |  |  |  |  |  |  |  |
| mtr-miR2629c | -0.007 | | 0.003 | | -0.012 | |  |  |  |  |  |  |  |  |  |  |  |  |
| mtr-miR2629b | -0.007 | | 0.003 | | -0.012 | |  |  |  |  |  |  |  |  |  |  |  |  |
| mtr-miR2629a | -0.007 | | 0.003 | | -0.012 | |  |  |  |  |  |  |  |  |  |  |  |  |
| hsa-miR-5004-3p | -0.010 | | -0.095 | | 0.276 | |  |  |  |  |  |  |  |  |  |  |  |  |
| mdo-miR-137b-5p | 0.021 | | 0.020 | | 0.100 | |  |  |  |  |  |  |  |  |  |  |  |  |
| bta-miR-2284ab | -0.106 | | -0.064 | | 0.058 | |  |  |  |  |  |  |  |  |  |  |  |  |
| oan-miR-1395-5p | 0.048 | | -0.067 | | -0.086 | |  |  |  |  |  |  |  |  |  |  |  |  |
| eca-miR-9080 | 0.111 | | 0.076 | | 0.122 | |  |  |  |  |  |  |  |  |  |  |  |  |
| cel-miR-2211-5p | -0.052 | | -0.216 | | 0.087 | |  |  |  |  |  |  |  |  |  |  |  |  |
| bdi-miR5065 | -0.158 | | 0.122 | | -0.140 | |  |  |  |  |  |  |  |  |  |  |  |  |
| dsi-miR-986-3p | -0.007 | | -0.116 | | -0.098 | |  |  |  |  |  |  |  |  |  |  |  |  |
| dme-miR-986-3p | -0.007 | | -0.116 | | -0.098 | |  |  |  |  |  |  |  |  |  |  |  |  |
| ath-miR5638a | -0.006 | | 0.090 | | -0.024 | |  |  |  |  |  |  |  |  |  |  |  |  |
| ame-miR-3741 | -0.099 | | 0.008 | | 0.032 | |  |  |  |  |  |  |  |  |  |  |  |  |
| ppy-miR-1273a | 0.100 | | -0.002 | | 0.045 | |  |  |  |  |  |  |  |  |  |  |  |  |
| dps-miR-2535-3p | -0.122 | | 0.226 | | 0.294 | |  |  |  |  |  |  |  |  |  |  |  |  |
| bdi-miR5065_1 | -0.137 | | 0.127 | | -0.143 | |  |  |  |  |  |  |  |  |  |  |  |  |
| mghv-miR-M1-2-3p | 0.071 | | -0.064 | | -0.024 | |  |  |  |  |  |  |  |  |  |  |  |  |
| dsi-miR-986-3p_1 | -0.013 | | -0.117 | | -0.096 | |  |  |  |  |  |  |  |  |  |  |  |  |
| dme-miR-986-3p_1 | -0.013 | | -0.117 | | -0.096 | |  |  |  |  |  |  |  |  |  |  |  |  |
| gga-miR-6608-3p | 0.070 | | -0.068 | | 0.080 | |  |  |  |  |  |  |  |  |  |  |  |  |
| csi-miR3953 | -0.234 | | 0.017 | | 0.014 | |  |  |  |  |  |  |  |  |  |  |  |  |
| cel-miR-8207-3p | -0.228 | | 0.161 | | 0.008 | |  |  |  |  |  |  |  |  |  |  |  |  |
| bmo-miR-3293 | 0.319 | | 0.042 | | -0.182 | |  |  |  |  |  |  |  |  |  |  |  |  |
| mmu-miR-6957-3p | 0.047 | | 0.026 | | -0.042 | |  |  |  |  |  |  |  |  |  |  |  |  |
| ppc-miR-83-5p | -0.558 | | 0.195 | | -0.016 | |  |  |  |  |  |  |  |  |  |  |  |  |
| ptc-miR6464 | -0.428 | | 0.076 | | -0.256 | |  |  |  |  |  |  |  |  |  |  |  |  |
| oan-miR-1421l-2-3p | -0.216 | | -0.782 | | 0.177 | |  |  |  |  |  |  |  |  |  |  |  |  |
| dme-miR-4949-3p | 0.059 | | -0.064 | | -0.641 | |  |  |  |  |  |  |  |  |  |  |  |  |
| cme-miR1863 | 0.122 | | 0.218 | | 0.350 | |  |  |  |  |  |  |  |  |  |  |  |  |

## Important microRNAs in PCA1 (feature selection)

## Important microRNAs in PCA2 (feature selection)

# (B) Clustering

## Amalgamation Steps

| **Step** | **Number of clusters** | **Similarity level** | **Distance level** | **Clusters joined** | | **New cluster** | **Number of obs. in new cluster** |
| --- | --- | --- | --- | --- | --- | --- | --- |
| 1 | 4 | 41.6956 | 26.0432 | 1 | 2 | 1 | 2 |
| 2 | 3 | 34.0068 | 29.4776 | 4 | 5 | 4 | 2 |
| 3 | 2 | 26.1549 | 32.9849 | 3 | 4 | 3 | 3 |
| 4 | 1 | 9.1639 | 40.5744 | 1 | 3 | 1 | 5 |
